# Placental Gene Therapy for Fetal Growth Restriction and Preeclampsia: Preclinical Studies and Prospects for Clinical Application

**DOI:** 10.3390/jcm13185647

**Published:** 2024-09-23

**Authors:** Sanjukta Majumder, Kristen Lee Moriarty, Youngmok Lee, Timothy M. Crombleholme

**Affiliations:** 1 Molecular Fetal Therapy Laboratory, Fetal Care Center at Connecticut Children’s Medical Center, Suite F254, 282 Washington Street, Hartford, CT 06106, USA; majumder@uchc.edu (S.M.); kmoriarty@uchc.edu (K.L.M.); 2 Fetal Surgery Section, Division of Pediatric General and Thoracic Surgery, Department of Surgery, UConn Health, Farmington, CT 06030, USA; 3 Division of Maternal-Fetal Medicine, Department of Obstetrics and Gynecology, Farmington, CT 06030, USA; 4 Department of Pediatrics, School of Medicine, University of Connecticut, Farmington, CT 06030, USA; yolee@uchc.edu

**Keywords:** IGF-1, gene therapy, placenta, fetal growth restriction, preeclampsia

## Abstract

In the last three decades, gene therapy has demonstrated significant progress. Over 700 active investigational new drug (IND) applications have been reported. Research on in utero gene therapy has advanced, but ethical and safety concerns persist. A novel approach under investigation is placental gene therapy, which holds promise for targeting diseases associated with placental dysfunction, such as fetal growth restriction (FGR) and preeclampsia. One of the underlying causes of placental insufficiency in these conditions is reduced placental growth factor-driven angiogenesis and endothelial cell dysfunction during fetal development. Studies have explored the overexpression of growth factor transgenes like IGF-1 to address FGR, yielding promising outcomes in animal models. Furthermore, intra-placental gene transfer, instead of systemic delivery of gene therapy vectors, has the potential to treat and cure these disorders. However, challenges and limitations akin to in utero gene therapy persist, including the risk of in utero infection, potential impairment of the mother’s future fertility, the risk of germline integration, and possible off-target effects of gene transfer in the fetus or the mother. Consequently, additional research and deliberation within the scientific and medical communities are warranted to fully comprehend the potential benefits and risks of placental gene therapy.

## 1. Introduction

Over the past three decades, the field of gene therapy has witnessed remarkable progress and significant advancements. There have been new and more efficient viral and non-viral vector systems, tissue-specific promoters and enhancers, and codon-optimized transgenes, with breakthroughs in the treatment of malignancies [1,2], metabolic deficiencies [3,4,5], genetic diseases [6,7,8,9,10], and blindness [11,12], to name a few. A recent joint statement from the US Food and Drug Administration and the National Institute of Health (NIH) reported that there are now over 700 active investigational new drug (IND) applications for gene therapy [13]. In utero gene therapy is an important goal in treating a broad spectrum of genetic, metabolic, neurologic, and hematopoietic conditions that incur significant morbidity and mortality before birth, which can only be addressed prenatally.

In 1999, Ismael Zanjani, PhD and W French Anderson, PhD first submitted two “pre-proposals” to the NIH Recombinant DNA Advisory Committee (RAC) for consideration. These were two concrete examples of in utero gene therapy to initiate discussion of the scientific, medical, ethical, and social issues inherent in prenatal application of this new technology [14]. At a minimum, they suggested that in utero gene therapy would only be appropriate for a disease “that is life-threatening or causes significant disability, either during or in early infancy, and it should be probable that based on the pathophysiology of the disease, treatment in utero would be beneficial and would not produce any significant risks to the fetus or the mother” [14]. The two pre-proposals on the treatment of adenosine deaminase-deficient severe combined immune deficiency (SCID) and the treatment of homozygous alpha-thalassemia were the topics of three consecutive RAC meetings [15]. The RAC working group recognized that prenatal gene transfer had remarkable potential for preventing and or treating serious and life-threatening genetic diseases. However, at that time, they concluded that there were insufficient preclinical data to pursue clinical trials. The RAC advisory committee recommended that there should be (1) accurate prenatal diagnosis of the condition to be treated; (2) provision of comprehensive information regarding the condition for the mother in her decision-making process; (3) the approach should minimize risk to the mother and fetus; (4) maximize the benefit to the fetus; (5) there should be negligible risk of germ line gene transfer; (6) it should only be indicated for diseases with serious morbidity or mortality prior to birth; (7) and there should be an absence of other severe abnormalities not corrected by gene delivery [16]. The RAC also recognized the need for technical advances in gene transfer that would allow for disease-specific transgene expression at high, durable, and regulatable expression. They also addressed safety aspects, including minimizing the risk of insertional mutagenesis, immune response to the transgene product, or the alteration in fetal development [17].

In light of the twenty-five years that have passed since the RAC advisory committee’s recommendations, the International Fetal Transplantation and Immunology Society (IFeTIS) recently reviewed these recommendations. They suggested additional criteria to address gaps that have emerged due to advances in the field [18]. The additional recommendations included (1) recognizing the importance of non-directive counseling, which is an essential component of the consent process in all fetal therapies; (2) the awareness that in utero gene therapy could covert a lethal condition to a survivable one, albeit with a severely disabled child with long-term morbidity; (3) the recognition of the well-established track record of safety of ultrasound-guided needle-based interventions which would be used for in utero fetal gene therapy; (4) risk of in utero infection; (5) risk of impaired future fertility of the mother; (6) risk of germline integration with gene transfer; (7) and the effects of off-target effect gene transfer in the fetus or the mother. However, neither the RAC advisory committee nor the IFeTIS recommendations considered the unique prospect of placental gene therapy.

## 2. Targeting the Placenta for Gene Delivery

The placenta is part of the fetoplacental unit, and systemic in utero gene therapy exposes the placenta to the gene therapy vector. Focusing the target of gene transfer solely on the placenta is a special circumstance. Many concerns noted for in utero gene therapy, such as accurate diagnosis, minimizing the risks to the fetus and the mother, negligible risk of germline gene transfer, and off-target effects of gene transfer vectors systemically administered, also apply to placental gene therapy.

The leading placental condition that would be amendable to placental gene therapy is fetal growth restriction (FGR), which complicates up to 10% of all pregnancies and is the second leading cause of morbidity and mortality [19]. The Society for Maternal Fetal Medicine currently estimates that 25–30% of all cases of FGR are due to placental insufficiency [19]; however, other reports estimate this to be up to 60% [20]. The pathophysiology of placental insufficiency in FGR is complex and multifactorial. An important component in the pathophysiology is a reduction in placental growth factor-driven angiogenesis and endothelial cell dysfunction [21]. Intra-placental gene transfer, as opposed to systemic presentation of the gene therapy vector, minimizes the risk of off-target gene transfer effects in the maternal–fetal dyad, and consequently, reduces the risk of germline gene transfer [22]. Unlike other indications for in utero gene therapy, a unique aspect of placental gene therapy is that the target organ for gene transfer is discarded at the end of gestation.

The utilization of placental gene therapy for placental disorders that affect fetal development, including fetal growth restriction (FGR), preeclampsia, and preterm birth, has advanced to specifically target the placenta, thereby mitigating some of the constraints associated with systemic fetal gene delivery. This review provides an overview of preclinical research in placental gene therapy. We focus on an analysis of the prospective indications and methodologies for clinical applications for placental gene therapy.

## 3. Placental Physiology and Placental Insufficiency

The placenta serves the role of providing nutrition, immunity, endocrine function, and oxygenation to the developing fetus during antenatal life. It also, as an organ itself, regulates homeostasis in the offspring. The formation of the placenta begins during embryonic life with the division of the blastocyst into trophoblast and invasion into the uterine decidua [23]. The trophoblast is further divided into cytotrophoblast and syncytiotrophoblast, which form the fetal component of the placenta or the chorion. The cytotrophoblast grows into the outer syncytiotrophoblast, forming the primary chorionic villi, with capillary beds feeding an open circulation bathing the villi from the maternal circulation of maternal spiral arteries. This allows nutrient and gas exchange at the cellular level, supporting the development of the fetus [24]. Proper invasion of the maternal spiral arteries from the trophoblasts is essential to allow a low-resistance circuit. Failure of vascular remodeling contributes to placental insufficiency, leading to impaired placentation. This can result in reduced oxygen and nutrient delivery to the fetus, which can cause acidosis and fetal hypoxemia [25].

Global placental dysfunction can impact the maternal–fetal dyad and induce metabolic changes that permanently affect the developing fetus [26]. Improper placental development and function are thought to be the fetal origin of adult diseases such as obesity, diabetes, and cardiovascular disease [27]. FGR has a significant impact on the growing fetus. Yet, there are currently no effective treatments to address placental insufficiency and prolong gestation. Current treatments are limited to delivery as a means of saving the fetus.

## 4. Placental Insufficiency in FGR

The placenta is essential for transferring nutrients, oxygen, and carbon dioxide between the mother and the fetus during pregnancy [28]. When placental insufficiency (PI) occurs, the placenta’s ability to transfer oxygen and nutrients to the fetus gradually fails to meet the needs of the developing fetus. In its most severe form, PI results in a state of decompensated hypoxia and acidosis. The reduced nutrient supply and low oxygen levels in fetal growth restriction (FGR) lead to a redistribution of blood flow, prioritizing blood flow to the baby’s brain at the expense of the body. This results in the recognizable clinical features of FGR [25,27].

Fetal growth restriction (FGR) is diagnosed when the estimated fetal weight (EFW) is below the 10th percentile for gestational age. It is further classified by severity. The Society for Maternal-Fetal Medicine (SMFM) defines severe FGR as less than the third percentile or, if abnormal Dopplers are present, prior to 32 weeks [19]. Additionally, FGR can further be divided into early-onset (<32 weeks) and late-onset (>32 weeks) [29]. In early-onset fetal growth restriction (FGR), placental dysfunction is usually more severe and may be indicated, although not always, by abnormal Doppler findings. Normally, the placenta exhibits low vascular resistance with forward flow in the umbilical arteries throughout the fetal cardiac cycle. However, in the case of early-onset FGR, which tends to be more severe, there is a deficiency in placental function, leading to increased cardiac afterload and high placental vascular resistance. This can result in absent or reversed diastolic umbilical artery flow, which in turn leads to a high pulsatility index in the ductus venosus and dilated cerebral vessels [21,29,30]. In later-onset FGR, the Doppler waveforms are usually preserved as they do not usually follow the same pattern as early-onset FGR [31]. Nevertheless, the most common cause of FGR is placental insufficiency (PI) due to failed vascular remodeling and endothelial cell dysfunction [32,33]. The possible introduction of placental gene therapy should target cases of early-onset FGR, which has the potential to dramatically improve perinatal outcomes.

## 5. Vectors for Placental Gene Therapy

The complexity of the fetal environment demands highly specific and regulated delivery platforms to minimize unintended effects on other tissues and maximize therapeutic efficacy. Additionally, the dynamics of the fetal environment, such as the pH sensitivity, cell turnover, and epigenetic modifications, further emphasize the need for optimized delivery vehicles for placental gene therapy [34].

Viral vectors, such as adenovirus, adeno-associated virus, retrovirus, and lentivirus have been modified to eliminate their pathogenicity. They are widely used as delivery systems in gene therapy for their high transduction efficiency and stable expression. However, each type of viral vector has unique challenges.

Adenoviruses have frequently been used in gene therapy. Yet, they may not be as suitable for in utero applications due to rapid cell division in the fetus, which quickly dilutes the episomal DNA. Adenoviruses are also highly immunogenic, which limits their utility for in utero applications. However, adenoviruses highly efficient gene transduction makes them excellent tools for proof-of-concept in utero studies in animal models [34,35].

Adeno-associated viruses (AAVs) are non-pathogenic in humans and elicit minimal immune response. AAVs have been used in gene therapy in over 200 clinical trials [36]. They have shown promise in in utero therapy for genetic disorders in animal models [37,38]. However, the small packaging capacity of AAVs limits the size of transgenes they can carry, and preexisting neutralizing antibodies against AAVs can reduce the effectiveness of the AAV vector. Evaluation is also needed to demonstrate AAVs’ efficacy and safety in the placental environment for placental gene transfer [34,39].

Retroviruses and lentiviruses have been studied for potential use in gene therapy. Concerns about oncogenicity due to insertional mutagenesis have limited application primarily to ex-vivo gene transfer. The development of self-inactivating lentivirus vectors has shown promise in treating immunodeficiencies and hemoglobin disorders [40,41]. Further studies are necessary to evaluate the safety and efficacy of these vectors for systemic in utero gene therapy in humans [34,42,43].

Due to the specific challenges viral vectors pose, non-viral vectors are becoming popular for gene therapy. Non-viral delivery methods include physical, inorganic, polymer-based, and lipid-based nanoparticles [34]. Inorganic nanoparticles, known for their biocompatibility and unique properties, hold potential in placental gene therapy. Yet, their effectiveness in placental gene therapy has been limited until recently [34]. Recent studies identified the potential for polymer-based nanoparticle-mediated delivery of IGF-1 to correct FGR in guinea pig models [34,44,45,46].

Gene editing technologies can potentially be combined to be used for placental gene therapy using any of these vectors. Gene editing permanently modifies the DNA sequence to rectify disease-causing mutations, insert normal genes, eliminate DNA sections, or silence disease-related genes. Technologies like CRISPR/Cas have advanced gene editing significantly since 2012 [47,48]. Precise delivery for fetal applications is crucial. If safely delivered, gene editing can potentially eliminate or correct pathogenic mutations early in development before irreversible pathology manifests. However, unlike fetus-targeting gene editing, which can permanently correct disease-causing mutations, it is unclear whether this approach will be beneficial for placental indications for gene therapy.

## 6. IGF-1 and Placental Insufficiency

Prior research has examined the impact of overexpressing growth factor transgenes that are believed to be crucial for placental growth and development and are deficient in PI. These include angiopoietin-1, angiopoietin-2, basic fibroblast growth factor (bFGF), insulin-like growth factor-1 (IGF-1), hepatocyte growth factor (HGF), placental growth factor (PlGF), platelet-derived growth factor-B (PDGF-B), and vascular endothelial growth factor (VEGF). Katz et al. studied adenoviral-mediated intra-placental gene transfer of eight different growth factor transgenes at embryonic day 14 (e14) in normal mice [49]. The rationale for this approach was that if a specific growth factor could enhance the structure and function of a presumably optimally functioning normal placenta, it would be an excellent candidate to test in models of placental insufficiency. They found that placentas harvested on e17 showed efficient gene transfer without fetal or maternal dissemination and that IGF-1, PDGF-B, and Ang-2 significantly increased placental morphometry without affecting fetal weight [49]. In addition, placental cross-sections following Ad-IGF-1 intra-placental gene transfer showed an expanded labyrinth area along both the maternal and fetal sides, with a significantly larger grid point count compared to controls. The surprising observation was that intra-placental gene transfer of these growth factors improved placental growth in normal mice, which were presumed to function at capacity. Despite the morphometric increases, the size of the newborn mice was not significantly increased compared to controls, suggesting other factors must influence the placental effect on fetal growth. This cross-sectional placental morphometry showed an increase in response to PDGF-B and Ang-2 expression, but the largest effect was observed with IGF-1, which significantly increased throughout the entire placenta compared to controls. In regard to the effect on survival, all dams survived except those treated with Ad-VEGF, in which the survival rate was only 40% [49]. Previous reports suggested that systemic Ad-VEGF is toxic at doses greater than 5 × 10^8^ PFU in non-pregnant age-matched mice [50]. These findings suggested that despite multiple growth factor transgenes increasing placental morphometry, IGF-1 may be the best available gene to test intra-placental injection in animal models of PI.

IGFs are synthesized in the placenta, particularly in the trophoblasts and endothelial cells. IGFs play a crucial role in the physiology of endothelial cells by enabling proliferation, migration, tube formation, and the production of vasodilator nitric oxide and vascular endothelial growth factor (VEGF) [51]. They also regulate cellular pathways such as protein synthesis and glucose metabolism [52,53]. IGF-1 enables angiogenesis via RAS/PI3K/IKK/NF-κB signaling pathways [51]. The placenta expresses IGF-1 receptors and IGF-1-binding proteins (IGFBPs) to regulate IGF-1’s functions. Research in multiple animal models and clinical FGR indicates that placental dysfunction reduces levels of insulin, IGF-1, and IGF-2, and elevates IGFBPs, further reducing their bioavailability. Not only is IGF-1 downregulated in FGR, but IGF-1 receptor (IGF1R) is also downregulated in FGR pregnancies [54,55,56,57,58]. Mice with null mutations in IGF-1 or IGF-2 have significantly reduced birth weight (60% reduction) compared to wild-type mice, and deficient IGF-1 also prevents VEGF-induced endothelial cell proliferation and survival [53].

Evidence suggests that IGF-1 triggers nuclear factor kappa B (NF-κB) signaling in endothelial cells and decreases apoptotic activation of NF-κB in postnatal settings [59,60,61,62,63]. Recent findings have shown that NF-κB signaling via an inhibitor of nuclear factor kappa-B kinase subunit beta (IKK-β) plays an essential role in fetal pulmonary endothelial cell growth and angiogenesis [64,65,66].

Other models, such as the hyperthermic sheep model of PI, have also been shown to be deficient in IGF-1. There is evidence of dysfunction in pulmonary artery endothelial cells with compromised angiogenesis in vivo, and proliferative potential and tube formation in vitro, which can be rescued by IGF-1 [66]. Endothelial-specific IKK-β-conditional-knockout pregnant mice are severely growth restricted [67]. Selective pharmacologic blockade of IKK- β replicates the in vitro signs of endothelial cell dysfunction [64]. However, it remains to be demonstrated how IGF-1 deficiency in PI results in IKK-β/NF-κB-dependent placental vascular endothelial cell dysfunction. We postulate that the lack of IGF-1-mediated NF-κB activation may cause endothelial cell dysfunction by impeding the expression of angiogenic genes, ultimately preventing placental development and fetal growth.

## 7. Placental Gene Therapy with Ad-IGF-1

**Figure 1:**
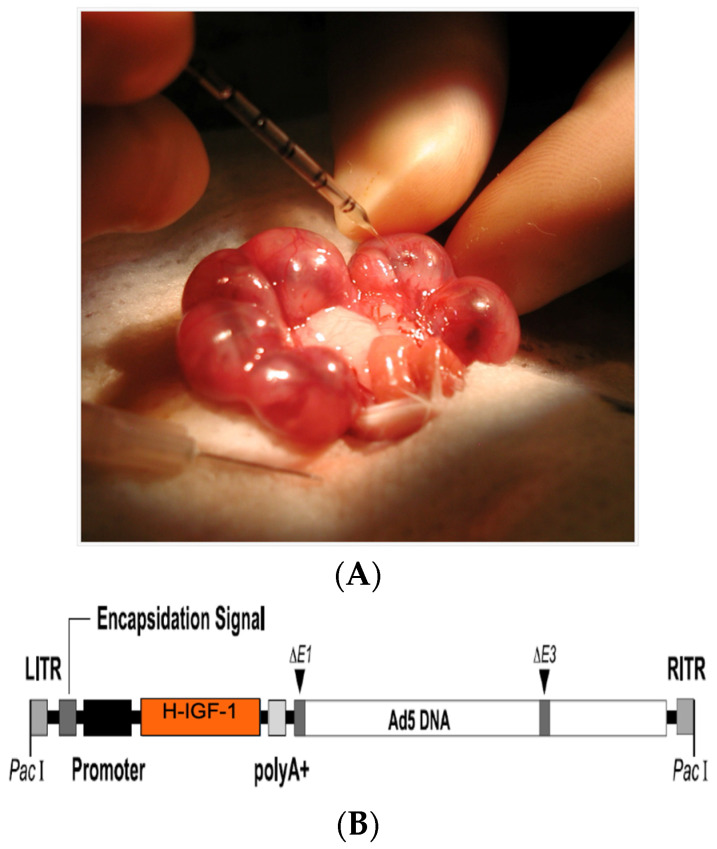
(**A**) Direct placental injection of Ad-5-hIGF-1 vector in the MUAL mouse model on e18 using a Hamilton syringe. (**B**) Construct of adenovirus-5 vector containing hIGF-1 gene used in placental gene therapy.

**Figure 2:**
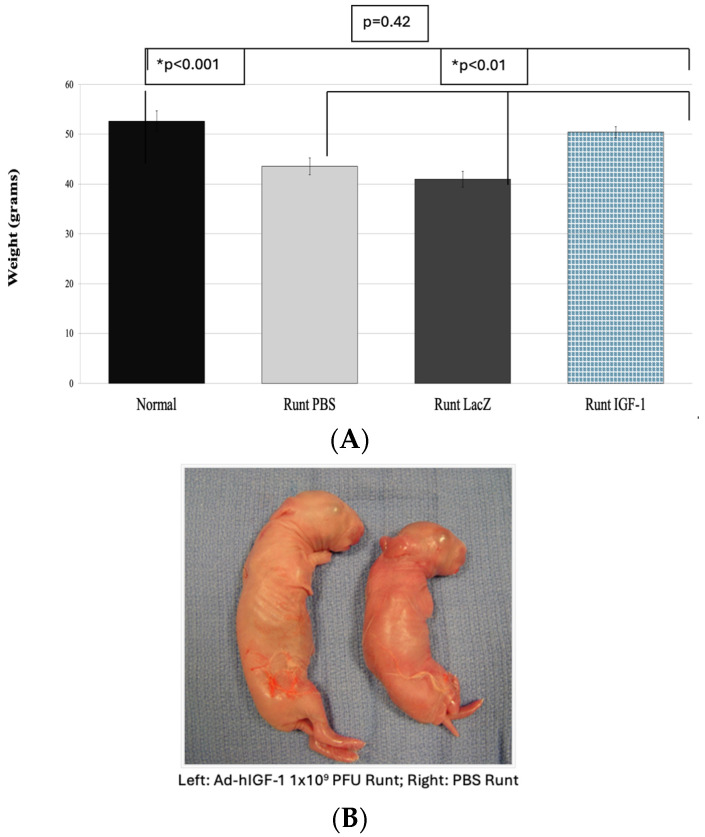
(**A**) Correction of fetal growth restriction in naturally occurring rabbit model of FGR using Ad-hIGF-1. (**B**) Gross specimen for comparison in pup weight between ad-hIGF-1 runt vs. PBS in rabbit runt model, * = significant.

Intra-placental adenoviral-mediated gene transfer of IGF-1 has shown promise in multiple animal models of PI, including the Wigglesworth model, the mesenteric uterine artery ligation model in mice, and the spontaneously occurring runt model in rabbits (Figure 1 and Figure 2) [68,69,70]. The Wigglesworth rat model of placental insufficiency is created by bilateral uterine artery ligation at the first position in time-mated Sprague Dawley rats on day 18 of a 22-day gestation. The first position, at the cervical end, and the last position, at the ovarian end, are excluded. This model reliably produces newborn rats that are significantly small for gestational age. Intra-placental gene transfer of adenovirus containing the human IGF-1 gene (Ad-hIGF-1, 1 × 10^9^ pfu) was performed to test its effect on fetal growth. The results showed that Ad-hIGF-1 corrected fetal birth weight in the growth-restricted pups, which was significantly different from sham-operated control pups at the same position in the opposite horn or those treated with phosphate-buffered saline (PBS) or Ad-LacZ (1 × 10^9^ pfu) reporter transgene [70].

To better reflect the features of clinical FGR due to PI, our laboratory developed the mesenteric uterine artery ligation (MUAL) model [71]. This model can be applied to transgenic mice to examine the role of specific genes in PI in response to Ad-IGF-1 (Figure 1A,B).

A total of 62% of mice that underwent MUAL developed a final weight < 10%, suggestive of FGR. They also had absent end diastolic flow in the umbilical artery, and a pattern of placental gene expression that mimicked human placental gene expression in FGR. The model was created by excluding the first and last positions and pups with a single mesenteric uterine artery. In pups supplied by two mesenteric uterine arteries, one was ligated, and the same position in the contralateral uterine horn was used as a control.

The limitations of the Wigglesworth and MUAL models of PI-induced FGR are that they are both surgical models and performed later in gestation. To test the effect of Ad-IGF-1 in a naturally occurring model of PI we turned to the runted model in rabbits. The rabbit has a bicornuate uterus, with each uterine horn containing four to six fetuses. Within both horns, there is a uterine vascular watershed area, located at the third position from the ovary, which reliably results in stunted growth (runt) with a term birth weight ratio of 0.85 compared to other fetuses. Rabbits have a longer gestation period compared to mice and rats, and the timing of this vascular insult more closely mimics mid-gestation FGR in humans [69].

Intra-placental injection of 1 × 10^9^ pfu of Ad-IGF-1 restored fetal birth weight and fetal, liver, and musculoskeletal weights to normal in the runted pups, with no change in the placental weight (Figure 2A,B).

Minimal gene transfer was detected by PCR in organs in the treated pups or maternal doe, suggesting that the off-target effects in the fetus and mother were rare and germline gene transfer was not observed. These findings suggest that intra-placental gene transfer could be performed with minimal risk of off-target gene transfer compared to systemic presentation of the vector. In addition, unlike in systemic in utero gene therapy, the target organ for gene transfer is discarded at the end of the gestation [69].

**Figure 3:**
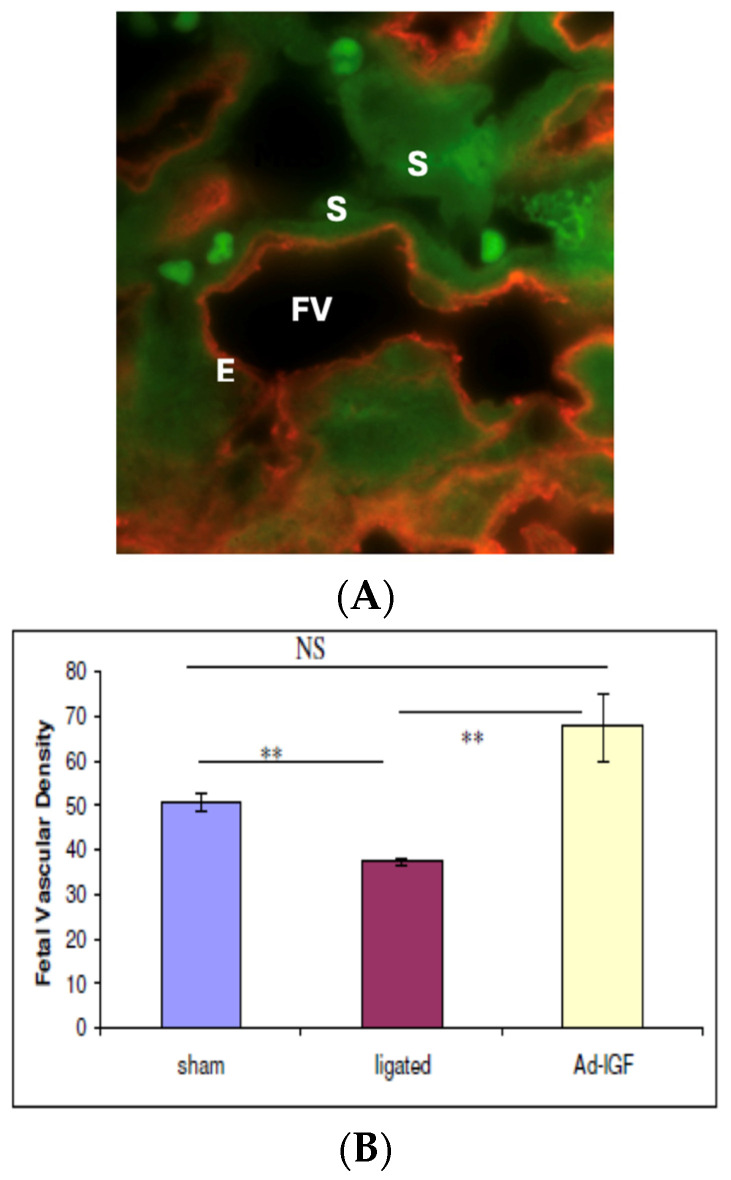
(**A**) High-power merged confocal image of placental tissue with fetal vessel (FV) section immunostained for fetal endothelial cells (E) (red) and syncytiotrophoblasts (S) (green), used for quantifying vascular density. (**B**) Effect of mesenteric uterine artery ligation (MUAL) and IGF-1 gene transfer on fetal microvascular density., NS = non-significant, ** = significant.

There may be multiple mechanisms by which IGF-1 gene transfer corrects PI and FGR due to the pleiotropic effects of IGF-1, which affect receptor expression, the function of glucose and amino acid transporters, and angiogenic gene expression. In vitro work by Jones et al. has demonstrated that IGF-1 increases glucose and amino acid transporter activity [68]. In addition, IGF-1 results in a significant increase in fetal microvascular density of the placenta and an expansion of endothelial progenitor cells [72]. The MUAL mice show a significant reduction in placental labyrinth depth, volume, and the level of IGF-1 and IGF-2 gene expression and a reciprocal increase in placental protein levels of IGFBP-2 and IGFBP-6 (Figure 3A,B). A single placental injection of 1 × 10^8^ pfu Ad5-hIGF-1 corrects placental insufficiency and FGR, normalizing birth weight compared to PBS and Ad-LacZ-tested control MUAL mice.

Other researchers, like de Vrijer et al., also found that in a hyperthermic sheep model of FGR, the decreased expression of placental IGF during early and mid-gestation could lead to placental insufficiency. This, in turn, might result in insufficient nutrient supply to the developing fetus later in gestation [57]. Research on restricted feeding during pregnancy in guinea pigs, which leads to FGR, revealed that undernutrition significantly negatively affects maternal circulating IGF-I and -II. This also resulted in increased levels of circulating IGFBPs during mid- and late pregnancy, further reducing the bioavailability of the IGFs. It was also linked to impaired fetal and placental growth during those stages of gestation [73].

## 8. Alternative Biomarkers for Potential Therapy Targets and Other Mechanisms and Vectors for Treating PI with Placental Gene Therapy

In addition to IGF-1, the development of the placenta relies on well-coordinated interaction of vascular growth factors and their receptors. This includes the involvement of vascular endothelial growth factor (VEGF) and placental growth factor (PlGF). VEGF and PlGF have been suggested to play a part in the vascular development of the placenta, with VEGF being responsible for angiogenesis and vasculogenesis, with importance in hypoxic conditions central to PI and overexpression noted in cancer [74].

An alternative approach for gene therapy in FGR was studied by David et al., using VEGF gene therapy from the maternal side through injection of the uterine artery as opposed to the fetal-placenta unit. Their research in sheep models has shown that mid-gestation injection of the Ad-VEGF-A165 into the uterine artery through laparotomy increased uterine artery blood flow and reduced short- and long-term vascular contractility [75]. In this FGR sheep model, uterine artery injection of the same dose of Ad-VEGF165 also significantly improved fetal growth in late gestation [75,76]. This work showed improvement in FGR. However, maternal vascular insufficiency is not the most common cause of PI, and systemic administration of VEGF165, regardless of vector, raises concerns about toxicity and maternal safety [77].

Brownbill and colleagues looked into transplacental gene therapy by administering Ad-VEGF-D across the human placenta. Their findings showed that Ad-VEGF perfusion did not change placental permeability or fetoplacental vascular resistance. However, there was a small increase in maternal LDH release. The study also found that there was limited transfer of the vector across the placental barrier, which emphasizes the safety of placental gene therapy [77].

Additionally, Stephens et al. developed a nanoparticle protocol for the induction of placental expression of human IGF-1 for the treatment of FGR in guinea pigs. On gestational day 30–33, the dams underwent transcutaneous placenta gene therapy with an injection of IGF-1 nanoparticle vs. a PBS sham. They found that liver weight as a percentage of body weight was reduced by maternal nutrient restriction and unchanged with IGF-1 nanoparticle administration. Expression of hypoxia-inducible factor 1 (Hif1α) and tumor necrosis factor (Tnfα) were also increased in the nutrient restricted mice vs. control but decreased in those treated with IGF-1 [78].

Jones et al. studied nanoparticle delivery of IGF-1 for the treatment of PI due to FGR. They delivered transgenes using a deblock copolymer (pHPMA-b-pDMAEMA) complexed to IGF-1 plasmid DNA. They found direct placental injection of PLAC-1hIGF-1 produces RNA expression and improves FGR [79].

## 9. Placenta Gene Therapy for Preeclampsia

Preeclampsia and hypertensive disorders of pregnancy present a major health impact to the maternal–fetal dyad, causing 2 to 8% of pregnancy-related conditions worldwide [80,81]. Preeclampsia has been linked to maternal multi-organ failure and coagulopathic processes. Exposure in utero to preeclampsia can have an impact on fetal growth and ultimately lead to them developing FGR [82]. Severe preeclampsia poses a risk for stroke, myocardial infarction, worsening cardiac dysfunction, kidney disease, and liver dysfunction [83]. Effective treatments for preeclampsia are limited, with aspirin prophylaxis used to decrease the onset and or development of the disease in at-risk mothers and delivery serving as the treatment of choice once the disease has developed [82].

Various pathophysiological mechanisms have been theorized to play a role in preeclampsia due to the diverse immunological, angiogenic, and metabolic changes that occur during disease onset. Cytotrophoblast differentiation into extravillous trophoblasts migrates to the maternal surface and replaces smooth muscle cells in the spiral arteries within the maternal decidua. This allows an interface for maternal–fetal oxygenation, which is thought to be aberrant in preeclampsia, leading to vascular remodeling and syncytiotrophoblast stress [84]. Typically, normal healthy placentas express placenta-like growth factor, which is deficient in placentas impacted by preeclampsia. Production of abnormal circulating angiogenic factors increases as a response to preeclampsia in the maternal circulation, such as soluble fms-like tyrosine kinase-1 (sFLT-1) and soluble endoglin (sENG), both of which may bind VEGF receptors, preventing their normal activation and limiting endothelial development, exacerbating placental ischemia. A healthy placenta secretes sufficient PlGF, a marker of placental health. In preeclampsia, higher levels of sFLT-1 compete with PlGF and VEGF for VEGF receptor binding sites, compromising angiogenic activity and causing endothelial cell dysfunction [83]. Studies have shown that the sFLT-1/PlGF ratio provides information about the patient before the onset of overt signs and symptoms [85]. Prior research has shown that injecting sFLT-1 into rodents induces a model of preeclampsia [86].

Various targets for placental gene therapy involving preeclampsia have been identified, such as utilizing viral vectors for targeted delivery of VEGF as well as non-viral vectors using nanoparticles [83]. Prior studies have shown that subcutaneous treatment with VEGF121 at 400 μg/kg twice a day for six days in a rodent model of sFlt-1-induced preeclampsia led to improvement in the preeclampsia phenotype, with a significant reduction in blood pressure and proteinuria by 72% compared to controls [87]. Furthermore, kidney damage induced by sFlt-1 overexpression was analyzed, and glomerular histology significantly improved with VEGF121 treatment [87].

Similar to its pro-angiogenic effects in placental gene transfer in FGR, we speculate that placental gene therapy with IGF-1 may address the underlying vascular endothelial cell dysfunction in preeclampsia. The combination of preeclampsia presenting with severe FGR at earlier gestational ages may share a common cellular pathophysiologic pathway through deficiency of IGF-1, which is in turn associated with reduced VEGF [83]. Early-onset preeclampsia, less than 34 weeks, may be an ideal target for placental gene therapy, and often occurs with severe FGR.

## 10. Initial Challenges and Limitations with Placental Gene Therapy

Significant advances in the field have occurred since the first pre-proposals submitted to the RAC by Drs. Zanjani and Anderson. The nature of the viral vectors, particularly AAV, provides a much safer means of transgene delivery, lowering the risk of insertional mutagenesis, immune response, and duration of transgene expression [88].

Yet multiple challenges must first be overcome for placental gene therapy to be considered for clinical application. Not only are there technical challenges with vector delivery, but potential risks to the maternal–fetal dyad, ethical concerns, regulatory hurdles, and a cost burden inherent in such experimentation remain.

The initial challenges that impact gene delivery center around finding an appropriate animal model for human correlation to allow eventual clinical trials. Additionally, selecting the target organ for fetal delivery depends on the disease being treated. There are also challenges regarding vector selection, with integration or non-integration into the host genome, and timing of vector injection at the appropriate gestation to mitigate downstream effects. Moreover, prior early genetic testing limitations prevented the detection in utero of rare diseases, but now, with rapid advances in next-generation sequencing technologies, monogenetic disorders are being diagnosed early in gestation. Finally, additional challenges revolve around our knowledge of the fetal immune system, which was thought to be immune deficient, but now, recent studies have shown the immunological potential of the fetus [89,90].

The risks of placental gene therapy involve treating two patients simultaneously with the overall goal of minimization of the risks to both. There is a conflict in performing gene transfer to benefit the fetus while managing the possible adverse reactions in the mother. The maternal immune system can hinder transgene expression with robust activation of both humoral and cellular-mediated immunities. Sensitization of the maternal immune system to transgenic cells may also impact the fetal outcome. Not only are there risks with germline gene transfer, but there is also the potential for insertional mutagenesis. This was seen in the prior ex vivo trials with retroviral gene therapy for the treatment of children with X-linked severe combined immune deficiency, with 5 of 20 children developing leukemia [91].

## 11. Ethical and Political Considerations for Placental Gene Therapy

There are notable ethical concerns with placental gene therapy that have hindered its development and slowed the progression of its application in clinical trials. The ethical debate surrounding the treatment of an unborn fetus with the risk of fetal harm and potentially causing periviable and/or preterm delivery is of concern. Moreover, treating a fetus has ethical implications with the lack of rights of the unborn fetus, with decisions being made by the mother by proxy [92]. Additionally, the idea of fetal gene therapy imparts risks to the mother, with psychological, emotional, and health concerns being paramount. Offering a potential cure to an otherwise incurable disease and or furthering life with possible concerns about the future quality of life after delivery remains an issue. The inclusion and exclusion criteria for patients participating in fetal gene therapy are important as there is a social dilemma for fetuses that parents may deem normal when society may find said fetuses abnormal, as in the example of a trisomy 21 fetus [93].

To mitigate risks to the maternal–fetal dyad, regulating governing bodies that exist to oversee human clinical trials may one day allow the introduction of placental gene therapy. However, a tremendous amount of regulatory oversight and protections are in place for pregnant women participating in randomized clinical trials. Fetal surgery is one aspect of fetal therapy in which many procedures are now deemed “standard of care”. The introduction of fetal or placental gene therapy into clinical practice of fetal therapy requires another layer of regulation provided by the Food and Drug Administration [93].

Lastly, there remains a financial component to fetal gene therapy. Gene therapy is currently extremely costly, potentially introducing a significant disparity within the population regarding access. Using such techniques favors patients with access to large-scale academic medical centers with complex coordinated care. It limits widespread opportunities to patients with lower socioeconomic statuses, calling into question the social justice of equal access to care.

## 12. Conclusions

Advances in gene therapy are broadening the scope of treatable conditions to encompass metabolic disorders, hematologic diseases, and neurodevelopmental disorders. Gene therapy may be leveraged to combat conditions such as fetal growth restriction and preeclampsia by correcting endothelial cell dysfunction and enhancing placental function. Progress from successful preclinical studies to human clinical trials depends on a strong focus on establishing safety and efficacy across diverse populations while navigating regulatory challenges to ensure that gene therapies meet stringent safety standards. This effort involves continuous collaboration between researchers, clinicians, and regulatory bodies [94].

The development of robust ethical guidelines is imperative to tackle concerns associated with gene therapy during pregnancy, including potential long-term effects on the child and societal impacts. Public engagement is key in educating and fostering trust in gene therapy technologies, dispelling misconceptions, and enabling informed decision making among potential patients and healthcare providers [92,95,96].
